# The relationship between sexual function and mental health in Iranian pregnant women during the COVID-19 pandemic

**DOI:** 10.1186/s12884-021-03812-7

**Published:** 2021-04-26

**Authors:** Fatemeh Effati-Daryani, Shayesteh Jahanfar, Azam Mohammadi, Somayeh Zarei, Mojgan Mirghafourvand

**Affiliations:** 1 Reproductive Health Research Center, Midwifery Department, Faculty of Nursing and Midwifery, Urmia University of Medical Sciences, Urmia, Iran; 2 Department of Public Health and Community Medicine, School of Medicine, Tufts University, Boston, USA; 3 Nursing and Midwifery Care Research Center, Department of Midwifery and Reproductive health, School of Nursing and Midwifery, Tehran University of Medical Sciences, Tehran, Iran; 4 Department of Midwifery, Shohada Hospital, Qom University of Medical Sciences, Qom, Iran; 5 Social Determinants of Health Research Center, Department of Midwifery, Tabriz University of Medical Sciences, P.O. Box: 51745-347, Shariati Street, Tabriz, 513897977 Iran

**Keywords:** Sexual function, Stress, Anxiety, Depression, COVID-19

## Abstract

**Background:**

Sexual function, a significant contributor to quality of life, is affected by various factors, including overall mental health. COVID-19 is a current pandemic that influences the mental health of various populations, especially pregnant women. Despite the importance of sexual health, the specific nature of its relationship to overall mental health during the COVID-19 pandemic is not clearly defined. Thus, this study investigates the relationship between sexual function and mental health during the COVID-19 pandemic in Iranian pregnant women.

**Methods:**

This descriptive-analytical, cross-sectional study was carried out among 437 pregnant women using the sociodemographic and obstetrics characteristics questionnaire, Female Sexual Function Inventory, Stress, Depression, and Anxiety Scales. Random sampling was employed to select pregnant women who had a medical record in Health Centers of Tabriz city, Iran. The questionnaires were sent to the participants’ cell phones via WhatsApp or text messages, including links of questionnaires and the participants completed these questionnaires. Spearman correlation test was used to determine the relationship between sexual function and stress, anxiety, and depression. Generalized linear modeling was used to estimate each of the independent variables (sociodemographic characteristics, stress, anxiety, and depression) on the dependent variable (sexual function).

**Results:**

The mean (Standard Deviation) sexual functioning (total) score was 20.0 (8.50) from the available range of 2 to 36. The mean (SD) of depression, stress, and anxiety scale was 4.81 (5.22), 5.13 (4.37), and 7.86 (4.50) (possible score ranging from 0 to 21), respectively. Based on Spearman’s correlation test, there was a significant reverse correlation between the total sexual function score and stress, anxiety, and depression, indicating that all three variables negatively impacted sexual functioning. Variables such as mild stress, spouse type of job, sufficient household income, living with parents, higher marital satisfaction, and higher gestational age had a significant, positive impact on sexual function and could predict 35.8% of the variance model.

**Conclusions:**

Sexual functioning was significantly impacted by stress, anxiety, and depression – all of which are heightened during a pandemic. This topic warrants further study, and the general public should be educated on the protective influence of safe sex/intimacy on overall mental health.

**Supplementary Information:**

The online version contains supplementary material available at 10.1186/s12884-021-03812-7.

## Background

The COVID-19 virus has been associated with a rapid increase in cases, hospitalizations, and deaths worldwide [1]. Limited information is available regarding the impact of this virus in general and in pregnancy. However, there is available information about diseases associated with other pathogenic coronaviruses (i.e., Severe Acute Respiratory Syndrome (SARS) and Middle East Respiratory Syndrome (MERS)), which might be helpful in understanding Coronavirus’s impact during pregnancy – and on overall mental health and well being. COVID-19 is associated with various problems and symptoms, from mild and asymptomatic, such as common colds, to severe symptoms such as severe respiratory diseases [2]. The COVID-19 pandemic has resulted in many problems and stressors such as limitation of activities, fear of illness, losing relatives, life-threatening conditions, unemployment, a decrease in income, and separation from the family [3]. The psychosocial and economic implications of the current pandemic and its impact on collective, dyadic, and individual adjustment are expected to have deleterious collateral effects on general health (4) and place vulnerable populations at a greater risk psychological problems [4].

Mental health is a state of well-being that enables a person to recognize their abilities and cope with everyday life’s usual stresses [5]. Enjoying good mental health during pregnancy plays a crucial role in the progression of pregnancy and the fetus’s development [6]. Pregnancy is a critical period for women in which prenatal psychological distress can occur [7]. Further, unhealthy levels of anxiety and stress during pregnancy are related to pregnancy complications [8]. Documentation of COVID-19’s deleterious impact on mental health is already available, to some extent, in the health sciences literature [9, 10].

In contrast, adequate sexual functioning and intimacy are protective factors and often bolster mental health [11–13]. Sexual function is a process that involves various organs of the body and includes a woman’s ability to reach sexual arousal, orgasm, and feeling of satisfaction and enhances the quality of marital life [14]. Despite COVID-19 powerful effect on the overall quality of life, little information and attention are focused on maintaining sexual health, and there is a dearth of information on sexual health during COVID at this point [15]. A study in Chinese men and women showed that the outbreak of the COVID-19 impacted sexual health; with the increasing prevalence of the virus, sexual intercourse became less frequent [16].

Some studies have shown a relationship between sexual function and mental health [12]. Women who have active and satisfying sexual function have higher emotional satisfaction and mental health [17]. A review article indicated that there were many psychological and physiological health benefits associated with sexual activity. They showed that having vaginal sex improves people’s mental health with improved satisfaction, quality of communication, emotional awareness and integration, less depression and fewer suicide attempts, happiness, less psychoticism, neuroticism, and reduced pain and voiding symptoms [18].

An essential component of the health care of pregnant women during pregnancy is to assess sexual function [19] and mental health [20]. However, despite the importance and high prevalence of sexual dysfunction [21], most people do not address the problem due to embarrassment or lack of view it as a medical problem [22]. Based on the review carried out on the available texts, there was no available study examining the relationship between pregnant women’s sexual function and mental health (stress, anxiety, depression) during the COVID-19 pandemic. Therefore, due to the psychological and physiological health benefits of sexual health and likely positive effects on mental health [17], we conducted this research to determine the relationship between sexual function and mental health among Iranian pregnant women during the COVID-19 pandemic.

## Methods

### Study design and participants

The present study is a descriptive-analytical cross-sectional study that has been approved by the Research Ethics Committee of Tabriz University of Medical Sciences. The following are the study’s inclusion criteria: having a medical record in health centers of Tabriz city, being interested in participating in the research, possessing a cell phone, and healthy pregnancy. The study’s exclusion criteria are suffering from a mental disorder or having a mental disorder history, medical problems during pregnancy, and high-risk pregnancy.

### Sampling

In this study, 437 pregnant women were selected through cluster sampling method from health centers of Tabriz city. At first, about one fourth (22 centers) of the health centers of Tabriz city (80 centers) were randomly selected. Afterward, the sample size was determined for each center proportionally by the total sample size. The researcher extracted pregnant women’s phone numbers in each center via the Integrated Health System (SIB), and the women who meet the eligibility criteria were selected randomly from each center using www.random.org based on the determined sample size for each center. The researcher contacted the selected women, and the participants were assured of the purposes of the research and its confidentiality. The participants were asked to click on the links and complete the sociodemographic and obstetrics characteristics questionnaire, female sexual function index (FSFI), and stress, depression, and anxiety scale (DASS) questionnaire sent via WhatsApp or text messages. The telephone number used to send the link was the personal telephone numbers of pregnant women that had previously been used to provide counseling services to pregnant women and were registered in the SIB system. Also, these phone numbers were added to a WhatsApp group, and pregnant women could ask questions and problems related to their pregnancy by text message or voice to the experts of the medical center affiliated to Tabriz University of Medical Sciences-Iran.

### Data collection tools

In this study, the sociodemographic and obstetrics characteristics questionnaire, Female Sexual Function Index (FSFI), Depression, Stress, And Anxiety questionnaire (DASS-21) were used to collect data. English language versions of FSFI and DASS-21 are submitted as supplementary files (Additional files 1 and 2).

Sociodemographic and obstetrics characteristics questionnaire included questions regarding age, education, occupation, spouse’s age, education, occupation, sufficiency of monthly income for livelihood expenses, spouse’s support, marital satisfaction, and several pregnancies, gestational age, and fetus’s gender based on the sonographic patterns.

The 19-item Female Sexual Function Index (FSFI) measures women’s sexual function in 6 independent domains, including desire, arousal, lubrication, orgasm, satisfaction, and pain. In accordance with the instructions of the designer of the questionnaire, the results of each domain are calculated by summing item responses of each domain and multiplying by a domain factor (given that in the FSFI questionnaire, the items of each domain are different from other domains, at first, in order to balance the domains, the scores obtained from each domain was summed and multiplied by the factor number). The domain factors are 0.6 for desire, 0.3 for arousal, lubrication, and orgasm, 0.4 for satisfaction and pain. The score range determined for desire, arousal, lubrication, and orgasm subscales is 1–5, for pain is 0–5 and for satisfaction is 0 or 1 to 5. The number 0 indicates that the participant did not have sexual intercourse within the last 4 weeks. The total score is the sum of scores collected from 6 domains. The highest scores indicate the best sexual function. Based on the balanced domains, each domain’s highest score equals six and for full scale ranges between 2 and 36. The cut-off point for determining sexual dysfunction is 28. The scores under 65% for each domain are considered the dysfunction of that domain [23]. The validity and reliability of this questionnaire have been approved in Iran [24]. Fakhri et al. who examined the conceptual, cultural and linguistic compatibility of the FSFI, found that this questionnaire is conceptually compatible with Iranian culture and has sufficient validity and reliability [25].

The short version of the DASS questionnaire includes three 7-item subscales, i.e., stress, anxiety, and depression, which were employed in this research. In this questionnaire, the scores are based on the Likert scale from never (0) to very much (3), and the total score is not calculated. A higher score is an indication of a poorer status [26]. This questionnaire has been validated in Iran, and its reliability was reported as 0.73 for anxiety subscale, 0.81 for depression subscale, and 0.81 for stress subscale [27]. Its reliability in Tabriz’s pregnant women was calculated for depression, anxiety, and stress subscales as 0.80, 0.72, and 0.80, respectively [28].

### Sample size

Sample size based on the study of Nasiri et al. [29] and using GPOWER software and based on the correlation coefficient between a sexual function with three variables of stress (*r =* − 0.46), anxiety (*r =* − 0.18) and depression (*r =* − 0.44), alpha = 0.05 and power = 90% were calculated. The sample size calculated based on the anxiety variable was higher than the two variables of depression and stress (*n* = 258). According to cluster sampling and considering design effect = 1.5 and 10% attrition in the final sample size, 426 people were calculated. In the present study, 437 people were evaluated.

### Data analysis

Data were analyzed using SPSS software (version 25). The descriptive statistics, including frequency and percentage, mean, and standard deviation, were used to describe the sociodemographic and obstetrics characteristics, sexual function, stress, anxiety, and depression. The normality of the quantitative data was measured using Skewness and Kurtosis that sexual function was normal and stress, anxiety, and depression had not normal distribution.

Spearman correlation test was used to determine the relationship between sexual function and stress, anxiety, and depression. A general linear model (GLM) was then used to estimate the effect of each of the independent variables (sociodemographic and obstetrics characteristics, stress, anxiety, and depression) on the dependent variable (sexual function).

## Results

This study’s response rate was 85%; 650 pregnant women were investigated, and 512 women were considered eligible for receiving the link for the questionnaire, and among them, 437 pregnant women completed the questionnaire from late March 2020 to early April 2020.

The mean (standard deviation) age of participants was 29.7 (3.3) and their age range was 19 to 44 years. 62.2% of the participants had an academic education, and 81.5% were housewives. Approximately half of their spouses (53.1%) were between 30 to 35 years old, and approximately two-thirds (59.0%) had academic education. One fourth (25.6%) of the spouses were shop owners or self-employed. About one third (30.7%) of the participants reported that their monthly income is below the sufficient level. Less than half (45.8%) of the participants lived in their own houses. Most of them (71.6%) stated that they receive much or very much support from their spouses, and 73.7% had marital satisfaction. Approximately half of the participants were experiencing their first pregnancy, 53.3% were in the third trimester, and the fetal sex of half of them was identified as male in accordance with the ultrasound (48.3%) (Table 1).

**Table 1:** Sociodemographic and obstetrics characteristics of the pregnant women (*n* = 437)

Characteristic	N (%)	Characteristic	N (%)
**Age** (Year)		**Husband’s age** (Year)	
< 25	244 (55.8)	< 30	140 (32.0)
25–35	118 (27.0)	30–35	112 (25.6)
> 35	75 (17.2)	> 35	185 (42.3)
**Mean (SD)** ^**a**^	29.7 (5.5)	**Mean (SD)** ^**a**^	329 (7.6)
**Education level**		**Spouse’s education level**	
Secondary school	21 (4.8)	Secondary school	36 (8.2)
High school	39 (8.9)	High school	51 (11.7)
Diploma	105 (24.0)	Diploma	92 (21.1)
University	272 (62.2)	University	258 (59.0)
**Job**		**Spouse’s job**	
Housewife	356 (81.5)	Clerk	97 (22.2)
Employed	81 (18.5)	Worker	91 (20.8)
**Place of residence**		Shopkeeper	60 (13.7)
Personal	200 (45.8)	Other ^c^	189 (43.2)
Rental	172 (39.4)	**Number of pregnancy**	
Other ^b^	65 (14.9)	1	241 (55.1)
**Household monthly income for expenses**		2	147 (33.6)
Completely sufficient	45 (10.3)	≥3	49 (11.2)
Relatively sufficient	258 (59.0)	**Gestational age** (Week)	
Insufficient	134 (30.7)	< 14	38 (8.7)
**Spouse’s support**		14–28	166 (38.0)
Extremely high	121 (27.7)	> 28	233 (53.3)
High	192 (43.9)	**Fetal sex**	
Moderate	106 (24.3)	Female	160 (36.6)
poor	18 (4.1)	Male	211 (48.3)
**Marital life satisfaction**		Unknown	66 (15.1)
Extremely high	135 (30.9)		
High	187 (42.8)		
Moderate	98 (22.4)		
Poor	17 (3.9)		

^a^All data indicates number (percent), unless specified

^b^Others indicate residence in parents’ house, relatives’ house and corporate house

^c^Others includes occupations such as construction, painter, agriculture, etc. (3 cases were unemployed)

The mean (SD) of the total score of sexual function was 20.0 (8.50) from the obtainable range of 2 to 36., and the mean (SD) of the total score of depression, stress, and anxiety were 4.81 (5.22), 5.13 (4.37), 7.86 (4.50), respectively, out of the possible score ranging from 0 to 21. Based on Spearman’s correlation test, there was a significant reverse correlation between total score of sexual function and stress (*r =* − 0.203, *p* <  0.001), anxiety (*r =* − 0.166, *p* = 0.001), and depression (*r =* − 0.234, *p <*  0.001) (Table 2).

**Table 2:** Relationship between sexual function and depression, stress and anxiety in pregnant women referred to Tabriz health centers during the COVID-19 pandemic in Iran (*n =* 437)

Characteristic	M (SD) ^**a**^	Med (P25-P75) ^**b**^	Relation to sexual function	
			r	P^**c**^
**Depression (0–21)**	5.22 (4.81)	4.00 (2.00–7.00)	- 0.234	< 0.001
**Stress (0–21)**	5.13 (4.37)	7.00 (5.00–11.00)	- 0.203	< 0.001
**Anxiety (0–21)**	7.86 (4.50)	4.00 (2.00–7.00)	- 0.166	0.001
**Total score of sexual function (2.0–36.0)**	20.0 (8.50)	22.30 (12.50–26.60)	–	–

^a^Mean (Standard deviation)

^b^Median (Percentile25- Percentile75)

^c^Spearman correlation test

According to the unadjusted GLM, there was a significant relationship between the score of sexual function and stress, anxiety, depression, age, occupation, spouse’s age, spouse’s occupation, the sufficiency of income for the living costs, place of residence, spouse’s support, marital satisfaction, and gestational age (*P <*  0.05). Based on the adjusted GLM and by adjusting other variables, there was a significant relationship between sexual function and variables of stress, spouse’s occupation, income sufficiency for the living costs, place of residence, marital satisfaction, and gestational age (*P <*  0.05). The variables could predict 35.8% of the variance of sexual function score in pregnant women during the COVID-19 pandemic (Table 3).

**Table 3:** Relationship between sociodemographic characteristics and sexual function in Iranian pregnant women referred to Tabriz health centers during the COVID-19 pandemic (*n* = 437)

Characteristic	Unadjusted GLM^b^		Adjusted GLM^b^	
	β (95% CI^**a**^)	***p***-value	β (95% CI^**a**^)	***p***-value
**Stress** (References: Normal)	**–**	**–**	**–**	**–**
Mild	−6.57 (−8.85 to − 4.29)	< 0.001	− 3.32 (− 5.70 to − 0.94)	0.006
Moderete	−1.94 (− 3.98 to 0.09)	0.061	0.31 (− 2.28 to 2.90)	0.816
Severe	− 3.27 (− 6.06 to − 1.38)	0.002	0.71 (− 2.76 to 4.18)	0.687
**Anxiety** (References: Normal)	**–**	**–**	**–**	**–**
Mild	1.06 (− 1.19 to 3.31)	0.356	−0.19 (− 2.50 to 2.11)	0.870
Moderete	− 2.31 (− 4.81 to 0.19)	0.070	− 0.71 (− 3.30 to 1.89)	0.592
Severe	−2.39 (− 4.39 to − 0.39)	0.019	−0.81 (− 4.03 to 2.41)	0.622
**Depression** (References: Normal)	–	–	**–**	**–**
Mild	−0.77 (0–3.09 to 1.56)	0.517	− 0.28 (− 2.77 to 2.21)	0.824
Moderete	− 0.53 (− 3.26 to 2.19)	0.700	2.57 (− 0.87 to 6.01)	0.143
Severe	−7.26 (−10.12 to −4.39)	0.001	−3.06 (− 6.69 to 0.57)	0.098
**Age** (References: > 35)	**–**	**–**	**–**	**–**
< 25	5.22 (2.67 to 7.76)	< 0.001	1.98 (− 1.16 to 5.12)	0.217
25–35	1.06 (− 1.07 to 3.19)	0.330	−1.28 (− 3.68 to 1.12)	0.295
**Job** (References: Employed)	**–**	**–**	**–**	**–**
Housewife	−2.36 (− 4.41 to − 0.31)	0.024	− 1.90 (− 3.81 to − 0.01)	0.050
**Spouse’s age** (References: > 35)	**–**	**–**	**–**	**–**
< 30	4.11 (2.28 to 5.95)	< 0.001	0.64 (− 1.52 to 2.80)	0.560
30–35	2.18 (0.22 to 4.14)	0.029	−0.08 (− 2.20 to 2.04)	0.939
**Spouse’s job** (References: Clerk)	**–**	**–**	**–**	**–**
Worker	− 4.87 (− 7.25 to − 2.49)	< 0.001	− 5.55 (− 7.88 to − 3.22)	< 0.001
Shopkeeper	−5.20 (− 7.87 to − 2.52)	< 0.001	−2.79 (− 5.22 to − 0.35)	0.025
Others	−4.62 (− 6.66 to − 2.59)	< 0.001	− 5.03 (− 6.94 to − 3.11)	< 0.001
**Sufficiency of income for expenses** (References: Insufficient)		**–**	**–**	**–**
Completely sufficient	−2.24 (− 4.86 to 0.37)	0.092	− 3.78 (− 6.41 to − 1.15)	0.005
Fairy sufficient	− 7.79 (− 9.40 to-6.17)	< 0.001	−5.49 (− 7.10 to − 3.88)	< 0.001
**Place of residence** (References: Personal)	**–**	**–**	**–**	**–**
Rental	1.82 (0.12 to 3.51)	0.036	−0.04 (− 1.62 to 1.54)	0.959
Others	5.84 (3.51 to 8.16)	< 0.001	7.03 (4.69 to 9.39)	< 0.001
**Spouse’s support** (References: Moderate)	**–**	**–**	**–**	**–**
Extremely high	2.62 (0.50 to 4.74)	0.015	−1.02 (−3.79 to 1.75)	0.469
High	2.59 (0.68 to 4.50)	0.008	0.85 (−1.54 to 3.23)	0.485
**Marital life satisfaction** (References: Moderately)		**–**	**–**	**–**
Extremely high	4.64 (2.56 to 6.72)	< 0.001	3.39 (0.41 to 6.36)	0.026
High	2.58 (0.64 to 4.52)	0.009	0.23 (−2.25 to 2.72)	0.854
**Gestational age** (References: Thired Trimester)	**–**	**–**	**–**	**–**
First Trimester	−6.12 (− 8.99 to −3.25)	< 0.001	−4.43 (− 7.13 to −1.74)	0.001
Second Trimester	0.18 (−1.48 to 1.85)	0.828	0.60 (−1.00 to 2.21)	0.458

Adjusted *R*^*2*^ = 0.358; ^a^ 95% Confidence Interval; ^b^ General Linear Model

## Discussion

The present study is the first study to investigate the relationship between mental health and sexual function in the COVID-19 pandemic in Iranian pregnant women. The results demonstrated a significant reverse correlation between the total score of sexual function and stress, anxiety, and depression. Accordingly, by improving sexual function, stress, anxiety, and depression were decreased in pregnant women. Furthermore, variables such as mild stress, spouse employment, sufficient household income, living with parents, higher marital satisfaction, and higher gestational age had a significant relationship with the higher score of sexual function.

This study showed a significant reverse relationship between pregnant women’s sexual function and psychological characteristics (anxiety, stress, and depression). This study’s results are similar to the result of the study conducted by Rahimi et al. (2018) in Iran [30]. Furthermore, our study results are consistent with the results of the study carried out by Seven et al. (2015) [31], which found out that 77.6% of women suffering from sexual dysfunction during pregnancy had high levels of anxiety and depression. The results are also consistent with the research carried out by Chang et al. (2012) regarding the reverse relationship between depression during the beginning and end of the pregnancy and sexual function [32]. It should be noted that anxiety, stress, and depression affect pregnant women’s sexual function. Any decrease in the quality of sexual relationship during pregnancy leads to depression [33], which indicates the reciprocal relationship between these two variables.

One of the predicting variables of sexual function in this study was stress in pregnant women, which was consistent with the study conducted by Hajnasiri et al. (2018) that showed a significant relationship between stress and sexual function during pregnancy [29].

The economic and social factors such as the amount of income and the working hours are among the predicting variables of sexual satisfaction and marital adjustment [34]. These results are similar to our study results since, in our study, the spouse’s occupation, i.e., governmental employee and higher family income, resulted in improved sexual function. Employment in governmental jobs and being sure of the earnings lead to women’s assurance and improvement of their sexual function. So that there are positive relationships between work-life balance, job satisfaction and life satisfaction [35]. In addition, these results are consistent with the study results by Saberi et al. (2018) [36], which indicated that employment status and economic status affect sexual function.

Social support has a significant reveres effect on depression, and depression affects the sexual function, i.e., increased social support decreases depression and, consequently, enhances sexual function [36]. In this study, participants living in their parents’ houses had a better sexual function than the participants living in their own houses. The role of social support of family decreases the mental load of individuals and, as a result, improves the sexual function.

Another predictor of sexual function in pregnant women during the COVID-19 pandemic in the present study is marital satisfaction. This study’s results are consistent with the results of the study conducted by Witting et al. (2008) [37]. This study demonstrated that overall marital satisfaction improves sexual function in pregnant women and decreases sexual dysfunction. This relationship has also been seen in the study of Aliakbari Dehkordi et al. (2010) [38]. A good sexual relationship that could result in the spouses’ marital satisfaction has a great role in the family’s success and stability. In addition to a good sexual relationship, other factors can affect marital satisfaction, which can be investigated in future studies. Factors such as economic problems that families face during the epidemic of diseases and conflicts due to mothers’ employment during this period lead to their failure to invest in their spouse’s relationship. These factors can lead to stress, which can result in tension in couples’ relationships, and consequently, the marital relations will weaken, especially during pregnancy, and as a result, marital satisfaction will decrease and result in sexual dysfunction.

The gestational age is among other predicting variables of the sexual function. The present study results revealed the decrease of sexual function in women during the first trimester in comparison to the third trimester. In the first trimester during the COVID-19 pandemic, pregnant women were experiencing anxiety and stress of the changes due to pregnancy nausea and vomiting and complications of the first months of pregnancy and the crisis of COVID-19 pandemic, which resulted in the decrease of sexual function.

Although still gender differences and differences in attitudes among men towards their future wives and daughters are still prominent in Iran, the lives of Iranian women are very diverse and vary depending on the geographical and cultural conditions of their place of residence [39]. The impact of globalization is quite evident in the daily life of Iranian women. Also, after the modernizations, Iran has experienced significant changes following the Islamic Revolution, and its homogeneous masculine texture has been disrupted and new groups and classes have emerged. One of these groups whose traditional social base has undergone tremendous changes during modernization is women [40].

One of the strengths of the present study is its large sample size and random sampling that increased the generalizability of the study results. Also, this study’s cross-sectional nature is one of the limitations of the design that the relationships shown do not exactly indicate a causal relationship. Another limitation of this study is the use of a questionnaire that certain themes related to sexual function maybe do not fit in the questionnaire.

## Conclusions

According to the results of the present study, it seems that the mental health status of pregnant women, sexual function, and the relationship between them are not in good condition. On the other hand, due to the effect of sociodemographic and psychological factors such as stress on sexual function and possible effects of sexual function on other aspects of life, it is recommended that the stakeholders and relevant officials involved must take this subject into account when designing guidelines and instructions during special situations such as diseases pandemic, in order to reduce its consequences as much as possible.

## Supplementary Information

**Additional file 1.** Questionnaire. English language versions of Female Sexual Function Inventory (FSFI).

**Additional file 2.** Questionnaire. English language versions of Stress, Depression, and Anxiety Scales (DASS).

## Data Availability

The datasets used and/or analysed during the current study are available from the corresponding author (M. Mirghafourvand) on reasonable request.

## Abbreviations

FSFI: Female Sexual Function Inventory
DASS: Stress, Depression, and Anxiety Scales
SARS: Severe Acute Respiratory Syndrome
MERS: Middle East Respiratory Syndrome
SD: Standard Deviation
