# Supplementary material for: The relationship between sexual function and mental health in Iranian pregnant women during the COVID-19 pandemic
Source: BMC Pregnancy Childbirth. 2021 Apr 26;21:327. doi: 10.1186/s12884-021-03812-7 (PMC8072090; doi:10.1186/s12884-021-03812-7)
Supplement: Supplementary file 1 — Additional file 1. Questionnaire. English language versions of Female Sexual Function Inventory (FSFI). [file 12884_2021_3812_MOESM1_ESM.pdf]

FSFI SCORING APPENDIX

Question	Response Options
1. Over the past 4 weeks, how **often** did you feel sexual desire or interest?	5 = Almost always or always<br>4 = Most times (more than half the time)<br>3 = Sometimes (about half the time)<br>2 = A few times (less than half the time)<br>1 = Almost never or never
2. Over the past 4 weeks, how would you rate your **level** (degree) of sexual desire or interest?	5 = Very high<br>4 = High<br>3 = Moderate<br>2 = Low<br>1 = Very low or none at all
3. Over the past 4 weeks, how **often** did you feel sexually aroused ("turned on") during sexual activity or intercourse?	0 = No sexual activity<br>5 = Almost always or always<br>4 = Most times (more than half the time)<br>3 = Sometimes (about half the time)<br>2 = A few times (less than half the time)<br>1 = Almost never or never
4. Over the past 4 weeks, how would you rate your **level** of sexual arousal ("turn on") during sexual activity or intercourse?	0 = No sexual activity<br>5 = Very high<br>4 = High<br>3 = Moderate<br>2 = Low<br>1 = Very low or none at all
5. Over the past 4 weeks, how **confident** were you about becoming sexually aroused during sexual activity or intercourse?	0 = No sexual activity<br>5 = Very high confidence<br>4 = High confidence<br>3 = Moderate confidence<br>2 = Low confidence<br>1 = Very low or no confidence
6. Over the past 4 weeks, how **often** have you been satisfied with your arousal (excitement) during sexual activity or intercourse?	0 = No sexual activity<br>5 = Almost always or always<br>4 = Most times (more than half the time)<br>3 = Sometimes (about half the time)<br>2 = A few times (less than half the time)<br>1 = Almost never or never

7. Over the past 4 weeks, how **often** did you become lubricated ("wet") during sexual activity or intercourse?

0 = No sexual activity
5 = Almost always or always
4 = Most times (more than half the time)
3 = Sometimes (about half the time)
2 = A few times (less than half the time)
1 = Almost never or never

8. Over the past 4 weeks, how **difficult** was it to become lubricated ("wet") during sexual activity or intercourse?

0 = No sexual activity
1 = Extremely difficult or impossible
2 = Very difficult
3 = Difficult
4 = Slightly difficult
5 = Not difficult

9. Over the past 4 weeks, how often did you **maintain** your lubrication ("wetness") until completion of sexual activity or intercourse?

0 = No sexual activity
5 = Almost always or always
4 = Most times (more than half the time)
3 = Sometimes (about half the time)
2 = A few times (less than half the time)
1 = Almost never or never

10. Over the past 4 weeks, how **difficult** was it to maintain your lubrication ("wetness") until completion of sexual activity or intercourse?

0 = No sexual activity
1 = Extremely difficult or impossible
2 = Very difficult
3 = Difficult
4 = Slightly difficult
5 = Not difficult

11. Over the past 4 weeks, when you had sexual stimulation or intercourse, how **often** did you reach orgasm (climax)?

0 = No sexual activity
5 = Almost always or always
4 = Most times (more than half the time)
3 = Sometimes (about half the time)
2 = A few times (less than half the time)
1 = Almost never or never

12. Over the past 4 weeks, when you had sexual stimulation or intercourse, how **difficult** was it for you to reach orgasm (climax)?

0 = No sexual activity
1 = Extremely difficult or impossible
2 = Very difficult
3 = Difficult
4 = Slightly difficult
5 = Not difficult

13. Over the past 4 weeks, how **satisfied** were you with your ability to reach orgasm (climax) during sexual activity or intercourse?	0 = No sexual activity<br>5 = Very satisfied<br>4 = Moderately satisfied<br>3 = About equally satisfied and dissatisfied<br>2 = Moderately dissatisfied<br>1 = Very dissatisfied
14. Over the past 4 weeks, how **satisfied** have you been with the amount of emotional closeness during sexual activity between you and your partner?	0 = No sexual activity<br>5 = Very satisfied<br>4 = Moderately satisfied<br>3 = About equally satisfied and dissatisfied<br>2 = Moderately dissatisfied<br>1 = Very dissatisfied
15. Over the past 4 weeks, how **satisfied** have you been with your sexual relationship with your partner?	5 = Very satisfied<br>4 = Moderately satisfied<br>3 = About equally satisfied and dissatisfied<br>2 = Moderately dissatisfied<br>1 = Very dissatisfied
16. Over the past 4 weeks, how **satisfied** have you been with your overall sexual life?	5 = Very satisfied<br>4 = Moderately satisfied<br>3 = About equally satisfied and dissatisfied<br>2 = Moderately dissatisfied<br>1 = Very dissatisfied
17. Over the past 4 weeks, how **often** did you experience discomfort or pain during vaginal penetration?	0 = Did not attempt intercourse<br>1 = Almost always or always<br>2 = Most times (more than half the time)<br>3 = Sometimes (about half the time)<br>4 = A few times (less than half the time)<br>5 = Almost never or never
18. Over the past 4 weeks, how **often** did you experience discomfort or pain following vaginal penetration?	0 = Did not attempt intercourse<br>1 = Almost always or always<br>2 = Most times (more than half the time)<br>3 = Sometimes (about half the time)<br>4 = A few times (less than half the time)<br>5 = Almost never or never
19. Over the past 4 weeks, how would you rate your **level** (degree) of discomfort or pain during or following vaginal penetration?	0 = Did not attempt intercourse<br>1 = Very high<br>2 = High<br>3 = Moderate<br>4 = Low<br>5 = Very low or none at all

FSFI DOMAIN SCORES AND FULL SCALE SCORE

The individual domain scores and full scale (overall) score of the FSFI can be derived from the computational formula outlined in the table below. For individual domain scores, add the scores of the individual items that comprise the domain and multiply the sum by the domain factor (see below). Add the six domain scores to obtain the full scale score. It should be noted that within the individual domains, a domain score of zero indicates that the subject reported having no sexual activity during the past month. Subject scores can be entered in the right-hand column.

Domain	Questions	Score Range	Factor	Minimum Score	Maximum Score	**Score**
Desire	1, 2	1 – 5	0.6	1.2	6.0	
Arousal	3, 4, 5, 6	0 – 5	0.3	0	6.0	
Lubrication	7, 8, 9, 10	0 – 5	0.3	0	6.0	
Orgasm	11, 12, 13	0 – 5	0.4	0	6.0	
Satisfaction	14, 15, 16	0 (or 1) – 5	0.4	0.8	6.0	
Pain	17, 18, 19	0 – 5	0.4	0	6.0	
Full Scale Score Range				2.0	36.0	
