# Supplementary material for: The relationship between sexual function and mental health in Iranian pregnant women during the COVID-19 pandemic
Source: BMC Pregnancy Childbirth. 2021 Apr 26;21:327. doi: 10.1186/s12884-021-03812-7 (PMC8072090; doi:10.1186/s12884-021-03812-7)
Supplement: Supplementary file 2 — Additional file 2. Questionnaire. English language versions of Stress, Depression, and Anxiety Scales (DASS). [file 12884_2021_3812_MOESM2_ESM.pdf]

DASS 21

NAME ______________________ DATE _________

Black Dog Institute

Please read each statement and circle a number 0, 1, 2 or 3 which indicates how much the statement applied to you <u>over the past week</u>. There are no right or wrong answers. Do not spend too much time on any statement.

The rating scale is as follows:

0 Did not apply to me at all - NEVER

1 Applied to me to some degree, or some of the time - SOMETIMES

2 Applied to me to a considerable degree, or a good part of time - OFTEN

3 Applied to me very much, or most of the time - ALMOST ALWAYS

FOR OFFICE USE

		N	S	O	AA	D	A	S
1	I found it hard to wind down	0	1	2	3			
2	I was aware of dryness of my mouth	0	1	2	3			
3	I couldn't seem to experience any positive feeling at all	0	1	2	3			
4	I experienced breathing difficulty (eg, excessively rapid breathing, breathlessness in the absence of physical exertion)	0	1	2	3			
5	I found it difficult to work up the initiative to do things	0	1	2	3			
6	I tended to over-react to situations	0	1	2	3			
7	I experienced trembling (eg, in the hands)	0	1	2	3			
8	I felt that I was using a lot of nervous energy	0	1	2	3			
9	I was worried about situations in which I might panic and make a fool of myself	0	1	2	3			
10	I felt that I had nothing to look forward to	0	1	2	3			
11	I found myself getting agitated	0	1	2	3			
12	I found it difficult to relax	0	1	2	3			
13	I felt down-hearted and blue	0	1	2	3			
14	I was intolerant of anything that kept me from getting on with what I was doing	0	1	2	3			
15	I felt I was close to panic	0	1	2	3			
16	I was unable to become enthusiastic about anything	0	1	2	3			
17	I felt I wasn't worth much as a person	0	1	2	3			
18	I felt that I was rather touchy	0	1	2	3			
19	I was aware of the action of my heart in the absence of physicalexertion (eg, sense of heart rate increase, heart missing a beat)	0	1	2	3			
20	I felt scared without any good reason	0	1	2	3			
21	I felt that life was meaningless	0	1	2	3			
					TOTALS			

This document may be freely downloaded and distributed on condition no change is made to the content. The information in this document is not intended as a substitute for professional medical advice, diagnosis or treatment. Not to be used for commercial purposes and not to be hosted electronically outside of the Black Dog Institute website. www.blackdoginstitute.org.au

DASS Severity Ratings

The DASS is a **quantitative** measure of distress along the 3 axes of depression, anxiety[1] and stress[2]. It is not a categorical measure of clinical diagnoses.

Emotional syndromes like depression and anxiety are intrinsically dimensional - they vary along a continuum of severity (independent of the specific diagnosis). Hence the selection of a single cut-off score to represent clinical severity is necessarily arbitrary. A scale such as the DASS can lead to a useful assessment of **disturbance**, for example individuals who may fall short of a clinical cut-off for a specific diagnosis can be correctly recognised as experiencing considerable symptoms and as being at high risk of further problems.

However for clinical purposes it can be helpful to have 'labels' to characterise degree of severity relative to the population. Thus the following cut-off scores have been developed for defining mild/moderate/severe/ extremely severe scores for each DASS scale.

Note: the severity labels are used to describe the full range of scores in the population, so 'mild' for example means that the person is above the population mean but probably still way below the typical severity of someone seeking help (ie it does not mean a mild level of disorder.

The individual DASS scores do not define appropriate interventions. They should be used in conjunction with all clinical information available to you in determining appropriate treatment for any individual.

[1]Symptoms of psychological arousal
[2]The more cognitive, subjective symptoms of anxiety

DASS 21 SCORE

DEPRESSION SCORE	ANXIETY SCORE	STRESS SCORE

	Depression	Anxiety	Stress
**Normal**	0 - 4	0 - 3	0 - 7
**Mild**	5 - 6	4 - 5	8 - 9
**Moderate**	7 - 10	6 - 7	10 - 12
**Severe**	11 - 13	8 - 9	13 - 16
**Extremely Severe**	14 +	10 +	17 +

This document may be freely downloaded and distributed on condition no change is made to the content. The information in this document is not intended as a substitute for professional medical advice, diagnosis or treatment. Not to be used for commercial purposes and not to be hosted electronically outside of the Black Dog Institute website.
www.blackdoginstitute.org.au
